# Structural characteristics of humic acids derived from Chinese weathered coal under different oxidizing conditions

**DOI:** 10.1371/journal.pone.0217469

**Published:** 2019-05-31

**Authors:** Liping Zhou, Liang Yuan, Bingqiang Zhao, Yanting Li, Zhian Lin

**Affiliations:** Key Laboratory of Plant Nutrition and Fertilizer, Ministry of Agriculture and Rural Affairs / Institute of Agricultural Resources and Regional Planning, Chinese Academy of Agricultural Sciences, Beijing, China; Old Dominion University, UNITED STATES

## Abstract

Humic acids derived from Chinese weathered coal were oxidized with hydrogen peroxide (H_2_O_2_) under various conditions, and their chemical composition and structure were examined. The raw material humic acids (HA) and oxidized humic acids (OHAs) were characterized by elemental analysis and ultraviolet visible (UV-Vis), Fourier transform infrared (FTIR), and solid-state ^13^C nuclear magnetic resonance (NMR) spectroscopy. Our results show that aromatic functional groups accounted for more than 70% of the HA and OHAs and there were significant differences in their structures and compositions. Compared to the HA, the average H and N contents of the OHAs decreased by 5.15% and 2.52%, respectively, and the average O content of those of the OHAs increased by 5.30%. The hydrophobicity index (HI) of HA is higher than those of the OHAs. Importantly, in the hypothesis test between the properties and preparation conditions of humic acid using SPSS, the partial η^2^ of the temperature, hydrogen peroxide concentration, liquid-solid ratio, and time were 0.809, 0.771, 0.748 and 0.729, respectively; thus, among the preparation conditions, temperature is the most important factor affecting the humic acids properties.

## Introduction

Weathered coal is formed when near-surface or shallow-surface coal is exposed to physical and chemical weathering for a long period [1]. Due to the influence of long-term weathering, weathered coal has a high oxygen content and low calorific value. However, it is rich in humic substances and has a variety of functional groups, such as carboxyl, hydroxyl, phenolic hydroxy thiol groups, etc., resulting in a high capability to enhance bioactivity. For instance, weathered coal can be used as a good natural adsorbent because of its adsorption, complexation and exchange properties [2–5]. Furthermore, humic acids are economically important because of the abundant global reserves of weathered coal, i.e., approximately 100 billion tons in China alone [6]. The content of humic acids of weathered coal is greater than that of lignite and peat [7]. However, research and applications of humic acids have mainly focused on the humic acids from peat and lignite [8–12], only relatively few reports were on the humic acids derived from weathered coal. Humic acids from weathered coal may have good prospects and advantages, that are worthy of investigation [7].

Existing research has been directed at characterizing humic acids through their degradation into individual monomers using hydrolysis, reduction, oxidation etc [13–15]. Among all degradation methods, oxidation can primarily release phenolic compounds and degrade aromatic rings containing oxygen to increase the contents of products such as benzenecarboxylic acids, phenolic acids and aliphatic dicarboxylic acids [16]. Some research has also found that oxidation can increase the oxygen-containing functional groups such as hydroxyl, ketone, and carboxyl groups of humic acid [16–17]. Currently, there are various oxidation methods and aqueous hydrogen peroxide is a suitable oxidant for coal oxidation from an industrial technology viewpoint because it is commonly available and environmentally friendly [13,18]. Importantly, oxidation by hydrogen peroxide can increase the content of carboxyl groups, whose protons participate in ion exchange, and have potential for separation and extraction of metal cations [19]. Zherebtsov et al found that during oxidation by hydrogen peroxide, the decrease in aromatic content lowers the content of free radicals, and the number of oxygen-bearing groups increased [20]. Doskocil et al found that the hydrogen peroxide degradation of humic acids resulted in oxidation of aromatic structures and cleavage of aromatic units. A high content of short chain carboxylic acids was detected in which malonic acid and succinic acid were predominate [21]. However, these studies mainly focused on the detection of hydrophilic fractions by producing many kinds of molecules. Few studies have tried to research the composition and functional groups of the “core” of humic acids and then prepare the materials for further study.

The reactivity of humic acids is determined by their chemical composition, structure, molecular weight and other properties [22–23]. Humic acids are used in various industries: in chemical industry, the presence of carboxyl groups can promote ion exchange and complex formation as well as facilitate the separation and extraction of metal cations from different media [1,24–25]; in the medical industry, the phenolic and anthraquinone structures in humic acids molecules may be involved in the electron transport system of biological redox [26–27]; in agriculture, some research has suggested that functional carboxylic and hydroxylic groups and hydrophobicity could play a major role in determining the activity of humic substances [28–29].

The objectives of this work are twofold: (1) to obtain oxidized humic acids with different structures and compositions to create knowledge base for the investigation of suitable applications for these oxidized humic acids and (2) to promote the development of efficient utilization technologies for the weathered coal in producing value-added fertilizers and other chemicals.

## Materials and methods

### Materials

The weathered coal employed for research were extracted from weathered coal of Qipanjing (E 107°12′, N 39°21′; Ordos City, Inner Mongolia Autonomous Region, Northeast China). The samples were collected from coal powder pulverized to 80-mesh and placed into a plastic bag for use. The humic acids used in the present investigation are representative, and they were extracted and purified from the weathered coal following the IHSS (International Humic Substance Society) methodology (alkali extraction method) with some modifications with the help of a company [30]. The sampled region has a temperate continental climate. The extracted humic acids accounted for 50.40% of the weathered coal weight. The ash content was 19.21%. The humic acids were stored in a sealed plastic bottle to prevent absorption of moisture from the air. The weathered coal in our research had high degree of weathering and oxidation [31], making it similar to the weathered coal in Huolinhe [32].

### Experimental method

**Fig 1:**
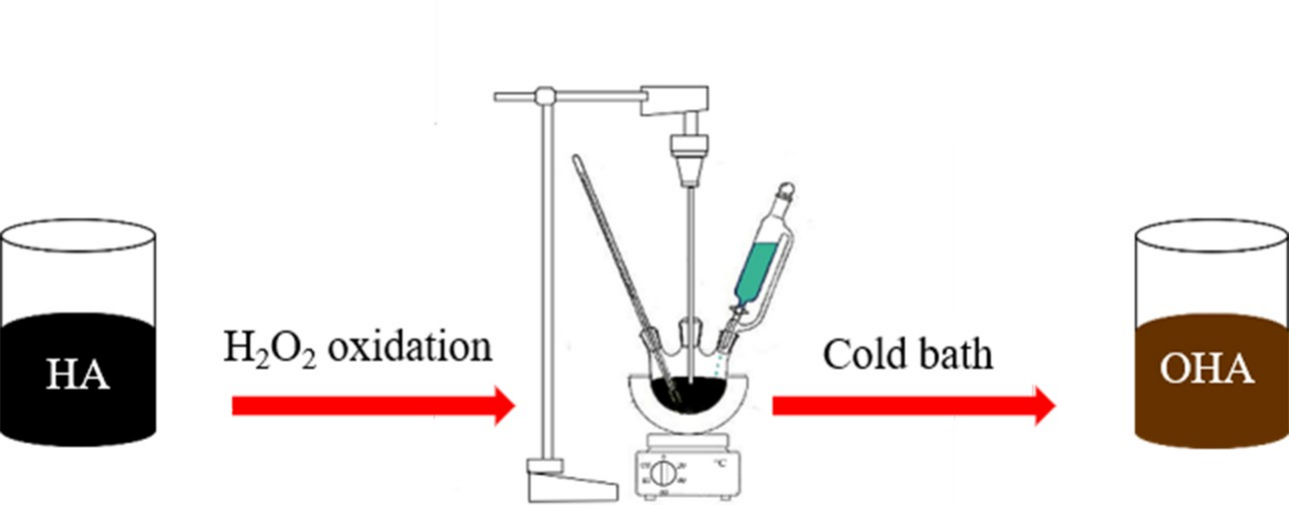
Schematic diagram of H_2_O_2_ oxidation of humic acids.

An orthogonal experimental design was applied to the humic acids oxidation experiment with four oxidation parameters at three different levels: the H_2_O_2_ concentrations of (5%, 10%, and 15%), liquid-solid ratio (0.5:1; 1.0:1; and 1.5:1), exposure time (1 h, 3 h, and 5 h) and temperature (40°C, 60°C, and 80°C). Samples subjected to the different oxidation parameters and levels are identified by the treatment code name shown in Table 1. Taking the preparation of OHA1 as an example, the details are as follows: 1 g of dried humic acids was dissolved in 10 mL water and mixed in an electric mixer to form a solution in a reactor. After heating the solution to 60°C, 0.5 mL H_2_O_2_ at concentration of 5% was instilled into the reactor under stirring conditions for 1 h to oxidize the humic acids. After the reaction completed, the reactor was immersed in an ice water at 0°C to quench the reaction. The process is shown in Fig 1 and oxidation conditions are tabulated in Table 1 together with the codes of the resultant OHAs. The prepared OHAs were freeze-dried and stored in a sealed plastic bottle.

**Table 1:** OHAs and oxidation conditions.

Treated Sample^1)^	Concentration of hydrogen peroxide	Liquid-to-solid ratio (mL/g)	Time (h)	Temperature (°C)
OHA1	5%	0.5:1	1	40
OHA2	5%	1.0:1	3	60
OHA3	5%	1.5:1	5	80
OHA4	10%	0.5:1	3	80
OHA5	10%	1.0:1	5	40
OHA6	10%	1.5:1	1	60
OHA7	15%	0.5:1	5	60
OHA8	15%	1.0:1	1	80
OHA9	15%	1.5:1	3	40

^1)^ HA, original humic acids which is derived from Chinese weathered coal; OHA1-OHA9, humic acids under different oxidation conditions.

### Humic acids characterization

The prepared OHAs and original humic acids were characterized by using an elemental analyzer and UV-Vis, FTIR and NMR spectroscopy to investigate the effect of different oxidation conditions on the structures and functional groups.

#### Elemental analysis

Elemental analysis was carried out with a Vario Micro Cube Elementar instrument (Elementar Analysensysteme GmbH, Germany). About 1mg dried humic acids was placed into the elemental analyzer to analyze the contents of C, H, N and O. The results were calculated as the molar ratios of C/N, C/H and O/C. The reference standards were acetanilide (C: 71.09%; N: 10.36%) and benzoic acid (H: 6.71%; O: 26.2%) to ensure the accuracy of the measurements. Each sample was measured three times.

#### UV-visible light scanning

The UV-Vis spectroscopy analysis of humic acids was performed by dissolving humic acids samples in a 0.05 M NaHCO_3_ solution (pH 8.3) to obtain a final concentration of 40 mg/L. UV-Vis spectra were obtained from 200 to 900 nm at room temperature with an Analytik SPECORD 200 PLUS UV/VIS spectrophotometer (Analytik Jena, Germany) at a scan speed of 600 nm min^−1^. The absorbance at 465 nm was divided by the value measured at 665 nm to determine the E4/E6 ratio coefficient, and the ratio of the absorbance of HA and OHAs at 280 and 360 nm was calculated as E2/E3 [33].

#### Fourier transform infrared spectroscopy

FTIR spectra were collected for random powder specimens dispersed in dried KBr pellets using a Bruker VERTEX 70 FTIR spectrometer. The pellets (2.0 mg of sample dispersed in 200 mg of KBr) were ground with an agate mortar before being pressed. FTIR spectra were recorded in the range of 4000–400 cm^-1^ with a 4 cm^-1^ resolution, and 64 scans were performed on each sample. To quantify the relative absorption intensity of each region of the carbon band, the spectra were baseline corrected and integrated with OMNIC 8.2 software. The major FTIR absorption bands and assignments are shown in Table 2.

**Table 2:** Major FTIR absorption bands and assignments for humic acids.

Frequency(cm^-1^)	Assignment
3450–3300	O–H stretching, N–H stretching (trace)
2940–2900	Aliphatic C–H stretching
1725–1720	C = O stretching of COOH and ketones (trace)
1660–1630	C = O stretching of amide groups (amide I band), quinone C = O and/or C = O of H–bonded C = O in conjugated ketones
1620–1600	Aromatic C = C, strongly H–bonded C = O of conjugated ketones
1590–1517	COO^−^symmetric stretching, N–H deformation + C = N stretching (amide II band)
1460–1450	Aliphatic C–H
1400–1390	OH deformation and C–O stretching of phenolic OH, C–H deformation of CH_2_ and CH_3_ groups, COO^−^antisymmetric stretching
1280–1200	C–O stretching and OH deformation of COOH, C–O stretching of aryl ethers
1170–950	C–O stretching of polysaccharide or polysaccharide-like substances, Si–O of silicate impurities
900–600	C–H surface deformation and vibration

#### Solid-state ^13^C–nuclear magnetic resonance spectroscopy

Solid-state ^13^C-CP/MAS–NMR spectroscopy was performed using a Bruker AVANCE III NMR 400 spectrometer (Bruker, Switzerland) [34–36]. A 4 mm magic angle spinning (MAS) probe was selected to determine the functional group assignments of the humic acids. Freeze-dried humic acids (approximately 100 mg) was packed into a zirconia rotor, and spectra was obtained by ^13^C cross-polarization/magnetic angle pinning (CP/MAS) NMR. The NMR measurements were carried out with the following parameters: temperature: 293.7 K, NMR-tube diameter: 4 mm, speed of spinning: 5 kHz, number of scans: 2048, CP time: 1 ms, ^1^H 90° pulse length: 4 μs, and recycle delay time: 0.8 s. The carbon-type content was determined by integration of the ^13^C NMR spectra according to the following chemical shift regions: alkyl C (C_Alk–H, R_): 0–45 ppm; methoxyl and N–alkyl C (C_Alk–O, N_): 45–60 ppm; O–alkyl C (C_Alk–O_): 60–91 ppm; di–O–alkyl C (anomeric) (C_Alk–di–O_): 91–110 ppm; aromatic C (C_Ar–H, R_): 110–142 ppm; O–aromatic C (C_Ar–O_): 142–156 ppm; carboxyl C (C_COO–H, R_): 156–186 ppm and carbonyl C (C_C = O_): 186–230 ppm [37]. The MestReNova 9.0.1 software was used for baseline correction and area integration.

### Statistical analyses

One-way analysis of variance (ANOVA) was used with Duncan’s test to evaluate significant differences (P < 0.05) in the elemental composition and the E2/E3 and E4/E6 ratios of the humic acids in SAS for Windows (Version 9.1). Principal component analysis (PCA) using the ^13^C–CP/MAS–NMR spectral data of the humic acids was performed using the software Canoco (version 4.5). A multivariate analysis of variance (MANOVA) was performed using SPSS (Version 20) to determine the relationship between the properties of humic acids and the preparation conditions.

## Results

### Changes in the elemental composition of the humic acids

Table 3 shows the elemental composition and atomic ratios of the HA and OHAs. The C, H, N and O contents of HA is 47.00%, 4.89%, 1.04% and 33.56%, respectively, and those of the OHAs varied, the C content was 44.96–50.61%, the H content was 4.20–4.97%, the N content was 0.90–1.10%, and the O content was 33.56–36.52%. Compared to those of HA, the average C, H and N contents of the OHAs decreased by 0.34%, 5.15% and 2.52%, respectively, and the average O content of the OHAs increased by 5.30%. The C content of OHA3 and OHA4, which were oxidized at 80°C, was higher than that of HA, and the contents of the other OHAs were lower than that of HA. The higher O content is obviously due to the oxidation process. OHA3 which was prepared at a hydrogen peroxide concentration of 5%, a liquid-to-solid ratio of 1.5:1, a reaction time of 5 h and a reaction temperature of 80°C, had the highest C content among all the samples, and OHA6 which was prepared at a hydrogen peroxide concentration of 10%, a liquid-to-solid ratio of 1.5:1, a reaction time of 1 h and a reaction temperature of 60°C, had the highest H and O contents. In this research, the average C/N, C/H and O/C ratios of the OHAs increased by 2.63%, 5.35% and 5.71%, respectively, relative to those of HA. OHA3 had the highest C/H ratio, and OHA6 had the smallest ratio. OHA6 showed C/H values of 0.78, whereas HA, OHA1, OHA2, OHA5, OHA7 and OHA8 had C/H values in the range of 0.80–0.84. OHA3, OHA4 and OHA9 which were prepared at 80°C and 40°C showed C/H values between 0.85–0.93.

**Table 3:** Elemental composition and atomic ratios of HA and OHAs under different oxidizing conditions.

Sample^1)^	Element content (%)				Atomic ratios			Ash contents
	C^2)^	H	N	O	C/N	C/H	O/C	
HA	47.00±0.22 c^3^^)^	4.89±0.05 ab	1.04±0.02 ab	33.56±0.12 e	52.97±1.09 b	0.80±0.01 bc	0.54±0.00 c	19.21±0.56 bcd
OHA1	45.86±0.10 cd	4.74±0.12 abcde	1.01±0.01 b	35.38±0.17 c	52.95±0.36 b	0.81±0.02 bc	0.58±0.00 ab	19.31±0.44 bcd
OHA2	46.29±0.22 cd	4.66±0.13 bcde	1.02±0.02 ab	35.87±0.24 b	52.94±1.17 b	0.83±0.02 bc	0.58±0.01 ab	18.22±0.78 de
OHA3	50.61±1.08 a	4.53±0.05 de	1.10±0.01 a	34.88±0.29 d	53.90±0.99 b	0.93±0.03 a	0.52±0.01 c	17.77±0.76 e
OHA4	48.48±2.60 b	4.81±0.23 abc	1.06±0.12 ab	35.15±0.06 cd	53.82±4.19 b	0.85±0.08 b	0.54±0.03 c	17.66±0.40 e
OHA5	45.69±0.23 cd	4.76±0.15 abcd	0.90±0.04 c	35.14±0.09 cd	59.51±2.55 a	0.81±0.02 bc	0.58±0.00 ab	18.54±1.28 cde
OHA6	46.57±0.45 c	4.97±0.01 a	1.01±0.03 b	36.52±0.49 a	53.79±0.93 b	0.78±0.01 c	0.59±0.01 a	19.76±0.69 bc
OHA7	46.42±0.39 cd	4.61±0.23 cde	1.04±0.02 ab	34.78±0.17 d	52.33±1.58 b	0.84±0.04 bc	0.56±0.01 b	19.89±0.18 ab
OHA8	44.96±0.17 d	4.49±0.12 e	0.90±0.07 c	34.79±0.25 d	58.45±4.95 a	0.84±0.02 bc	0.58±0.01 ab	18.71±0.88 bcde
OHA9	46.61±0.70 c	4.20±0.13 f	1.06±0.01 ab	35.52±0.11 bc	51.52±0.34 b	0.93±0.04 a	0.57±0.01 ab	20.99±0.08 a
OHA average	46.83	4.64	1.01	35.34	54.36	0.85	0.57	18.98

^1)^ HA, original humic acids which is derived from Chinese weathered coal; OHA1-OHA9, humic acids under different oxidation conditions.

^2)^ The mean of three analyses.

^3)^ Different lowercase letters in a column mean significant difference at the 5% level.

### UV-Vis spectra of the humic acids

The E2/E3 and E4/E6 ratios are tabulated in Table 4, and it is clear that both the ratios are higher for the OHAs than HA. The E2/E3 and E4/E6 values of HA were 1.57 and 3.10, respectively. The E4/E6 ratios of the OHAs were between 3.52 and 3.69, and the E2/E3 ratios of the OHAs were in the range of 1.88–1.97. Compared with HA, the average E2/E3 and E4/E6 ratios of the OHAs increased by 23.57% and 17.74%, respectively. The E2/E3 ratio of OHA1 and OHA8 was lower than that of the other OHAs. The E4/E6 ratios of OHA1, OHA3 and OHA8 was lower than that of the other OHAs.

**Table 4:** E2/E3 and E4/E6 ratios of OHAs and HA under different oxidizing conditions.

Sample^1)^	E2/E3^2)^	E4/E6
HA	1.57±0.02 c^3)^	3.10±0.02 c
OHA1	1.88±0.04 b	3.52±0.03 b
OHA2	1.97±0.06 a	3.68±0.01 a
OHA3	1.95±0.02 a	3.61±0.00 ab
OHA4	1.93±0.01 a	3.65±0.01 a
OHA5	1.95±0.01 a	3.68±0.01 a
OHA6	1.94±0.01 a	3.69±0.00 a
OHA7	1.94±0.01 a	3.67±0.02 a
OHA8	1.92±0.01 ab	3.59±0.01 ab
OHA9	1.93±0.01 a	3.64±0.01 a
OHA average	1.94	3.65

^1)^ HA, original humic acids which is derived from Chinese weathered coal; OHA1-OHA9, humic acids under different oxidation conditions.

^2)^ The mean of three analyses.

^3)^ Different lowercase letters in a column mean significant difference at the 5% level.

### Structural differences among the humic acids revealed by FTIR spectroscopy

**Fig 2:**
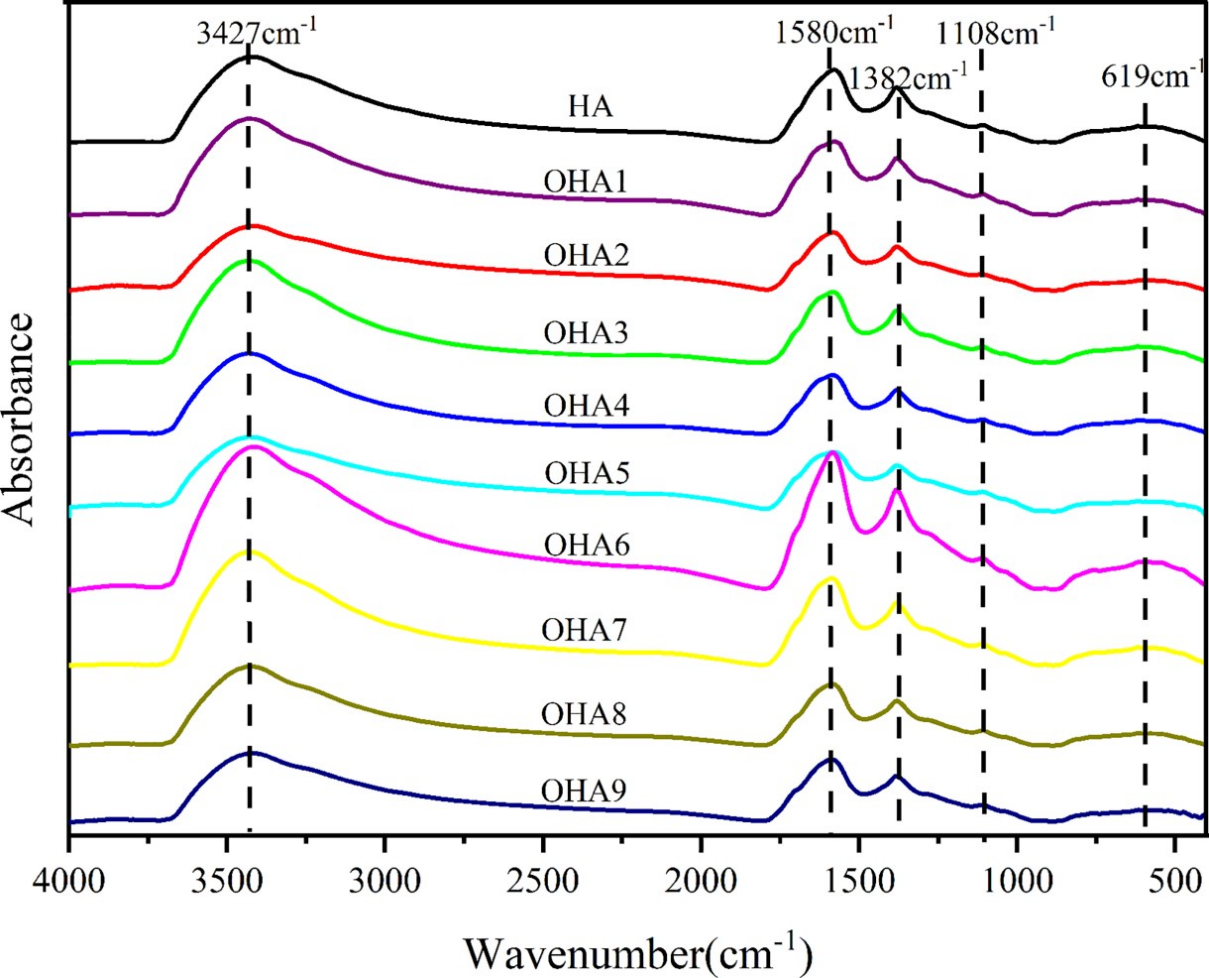
FTIR spectra of HA and OHAs under different oxidizing conditions. HA, original humic acids which is derived from Chinese weathered coal; OHA1-OHA9, humic acids under different oxidation conditions.

The FTIR spectra of the different humic acids are shown in Fig 2. The HA and OHAs have similar primary absorption bands as follows: (1) 3427 cm^–1^: broad absorption peak at 3500–3400 cm^–1^ due to C = C stretching in aromatic rings and O–H stretching in alcohol and phenol groups. (2) 1580 cm^–1^: peak due to COO–symmetric stretching, N–H deformation and C≡N stretching (amide Ⅱ band). (3) 1382 cm^–1^: peak indicating OH deformation and C–O stretching of phenolic OH, C–H deformation of CH_2_ and CH_3_ groups, and COO–antisymmetric stretching. (4) 1108 cm^–1^: peak due to C–O stretching of polysaccharide or polysaccharide-like substances and the Si–O in silicate impurities. (5) 619 cm^–1^: peak due to C–H surface deformation and vibration.

When comparing the relative absorption intensities (Table 5), the relative absorption peak of OHA3 at 3427 cm^–1^ is stronger than that of the other OHAs, and this peak is due to C = C stretching in aromatic rings and O–H stretching in alcohol and phenol groups. OHA6 had the weakest absorption peak at 3427 cm^–1^, and OHA3, OHA4 and OHA8, which were prepared at 80°C, had higher relative absorption intensities at 3427 cm^–1^ than the other OHAs, prepared at 40°C and 60°C, with the exception of OHA1. OHA2 which was prepared at 60°C had the strongest absorption peaks at 1580 cm^–1^, and OHA3 had the weakest absorption peaks at 1580 cm^–1^. Absorption in this area is attributed to COO–symmetric stretching, N–H deformation and C≡N stretching (amide Ⅱ band). OHA6 had the strongest absorption peaks at 1382 cm^–1^, and OHA5 had the weakest. The absorption in this area is due to OH deformation and C–O stretching of phenolic OH, C–H deformation of CH_2_ and CH_3_ groups, and COO–antisymmetric stretching. HA had the weakest absorption peak at 1108 cm^–1^, which is due to the C–O stretching of polysaccharides or polysaccharide-like substances and Si–O from silicate impurities. The absorption peaks at 1108 cm^–1^ for OHA5, OHA7 and OHA8 were the same as those prepared at 40°C, 60°C and 80°C, respectively. The absorption peak of OHA6 at 619 cm^–1^ was the strongest, followed by that of HA, and the peak at 619 cm^–1^ is due to C–H surface deformation and vibration.

**Table 5:** Relative absorption intensity of the FTIR spectra of the HA and OHAs.

Sample	Relative absorption intensity (%)				
	3427 cm^–1^	1580 cm^–1^	1382 cm^–1^	1108 cm^–1^	619 cm^–1^
HA^1)^	72.13	14.75	3.33	0.24	9.56
OHA1	73.45	14.89	2.85	0.36	8.45
OHA2	70.14	17.43	3.30	0.34	8.79
OHA3	74.67	13.71	3.01	0.40	8.21
OHA4	73.77	13.92	2.94	0.30	9.07
OHA5	71.44	16.09	2.78	0.31	9.39
OHA6	69.85	16.38	3.73	0.33	9.71
OHA7	73.30	14.83	3.07	0.31	8.50
OHA8	73.36	14.99	3.23	0.31	8.11
OHA9	72.32	17.29	3.50	0.36	6.53

^1)^ HA, original humic acids which is derived from Chinese weathered coal; OHA1-OHA9, humic acids under different oxidation conditions.

### Structural differences among the humic acids revealed by ^13^C–CP/MAS–NMR spectroscopy

**Fig 3:**
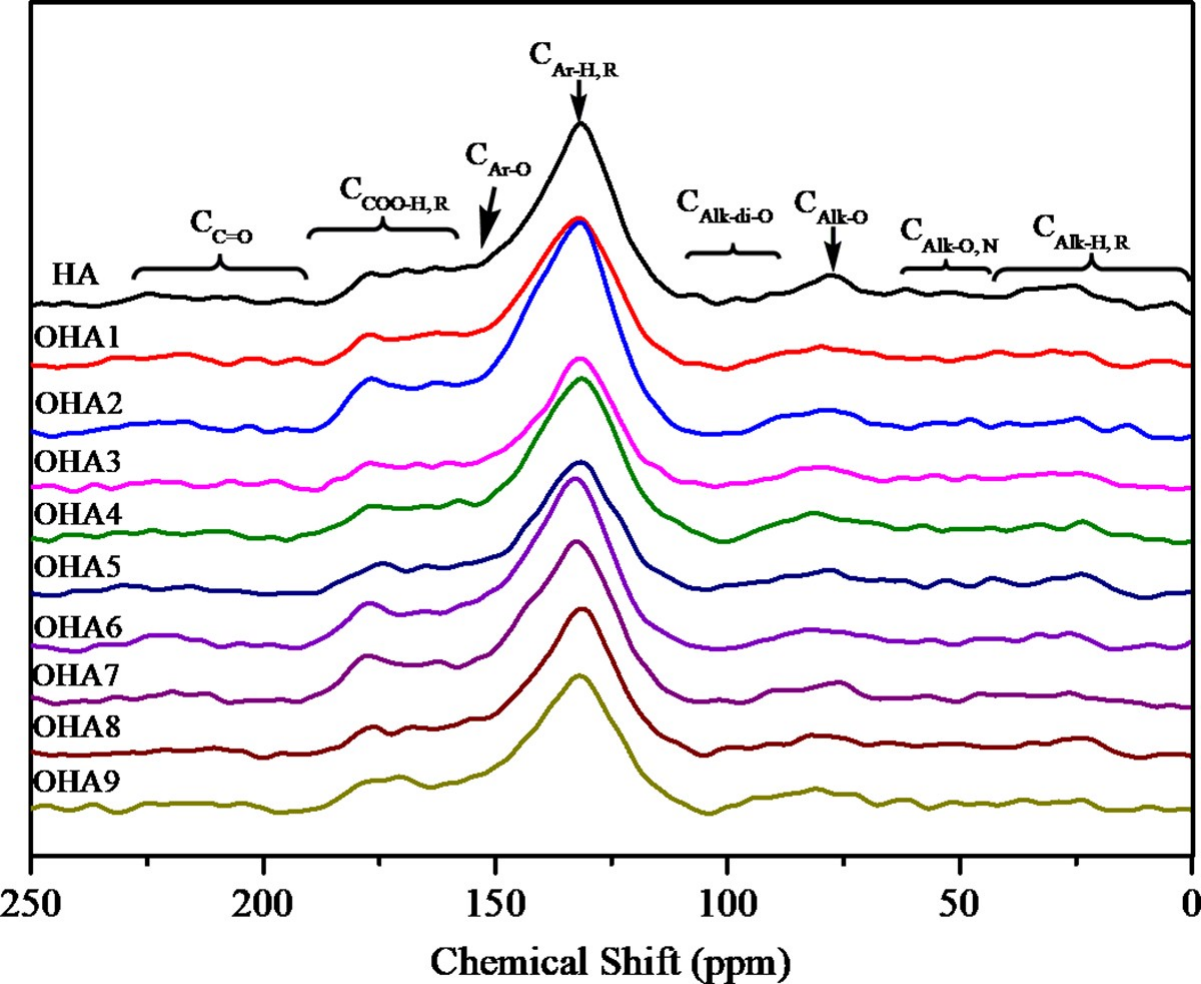
^13^C–CP/MAS–NMR spectra of HA and OHAs under different oxidizing conditions. HA, original humic acids which is derived from Chinese weathered coal; OHA1-OHA9, humic acids under different oxidation conditions.

**Fig 4:**
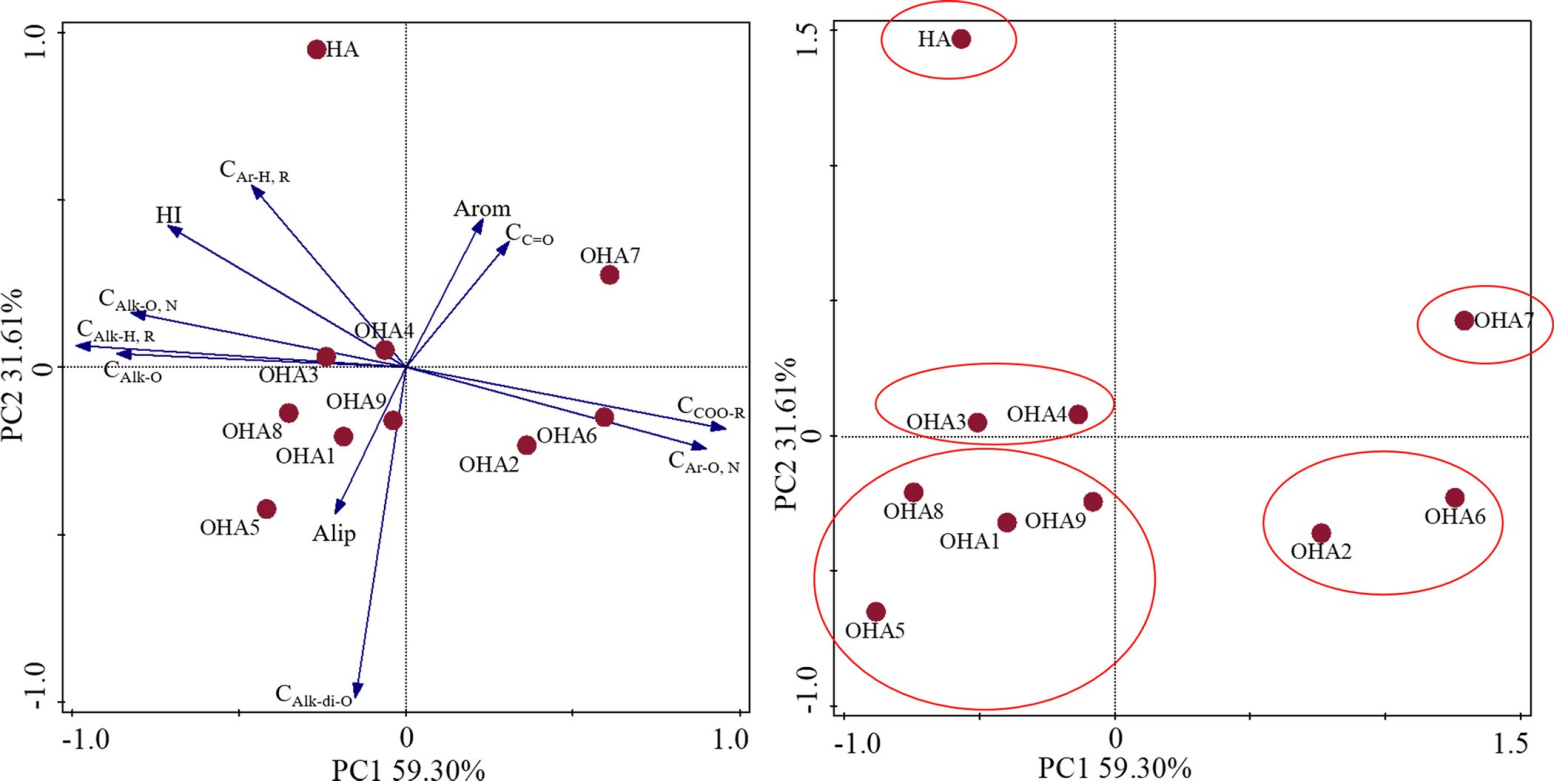
Principal components analysis (PCA) for the data obtained from the ^13^C–CP/MAS NMR spectra of the HA and OHAs. HA, original humic acids which is derived from Chinese weathered coal; OHA1-OHA9, humic acids under different oxidation conditions.

Solid ^13^C–CP/MAS–NMR spectra were obtained for HAs subjected to different treatments and are shown in Fig 3. The relative distribution of the signal areas for the different treatments is summarized in Table 6. The results showed that all the HA and OHAs contain alkyl, methoxyl, N–alkyl, O–alkyl, di–O–alkyl, aromatic, O–aromatic, carboxyl and carbonyl carbons. As shown in Fig 4, the highest abundance in all the spectra occurred in the chemical shift range of 110–142 ppm, which suggested that carbon was mainly present in the form of aromatic compounds. The aromatic functional groups accounted for more than 70% of the carbon composition when the O–aromatic carbon was also taken into consideration. Additionally, the second most abundant functional group, which was in the chemical shift range of 156–186 ppm, was carboxyl carbon, which accounted for approximately 15%. The other functional groups accounted for less than 15% of the total carbon composition.

**Table 6:** Relative distributions (percentages) of carbon types in the ^13^C–CP/MAS–NMR spectra.

Sample^1)^	C_Alk–H, R_	C_Alk–O, N_	C_Alk–O_	C_Alk–di–O_	C_Ar–H, R_	C_Ar–O, N_	C_COO–H, R_	C_C = O_	Arom^2)^	Alip^3)^	HI^4)^
HA	0.036	0.024	0.078	0.001	0.611	0.134	0.132	0.016	0.744	0.256	3.541
OHA1	0.033	0.009	0.061	0.011	0.588	0.151	0.149	0.020	0.739	0.261	3.234
OHA2	0.004	0.012	0.051	0.009	0.578	0.167	0.187	0.010	0.745	0.255	2.972
OHA3	0.028	0.019	0.076	0.006	0.579	0.142	0.139	0.012	0.72	0.280	3.301
OHA4	0.016	0.016	0.079	0.005	0.611	0.140	0.150	0.008	0.751	0.249	3.241
OHA5	0.043	0.019	0.092	0.018	0.562	0.133	0.141	0.009	0.695	0.305	2.907
OHA6	0.002	0.007	0.053	0.007	0.558	0.169	0.184	0.016	0.727	0.273	2.832
OHA7	0.003	0.004	0.049	0.002	0.562	0.166	0.202	0.015	0.728	0.272	2.732
OHA8	0.036	0.022	0.072	0.009	0.585	0.146	0.138	0.008	0.731	0.269	3.475
OHA9	0.018	0.009	0.079	0.008	0.569	0.151	0.159	0.008	0.719	0.281	2.948

^1)^ HA, original humic acids which is derived from Chinese weathered coal; OHA1-OHA9, humic acids under different oxidation conditions.

^2)^ Aromaticity: Arom = ([C_Ar–H, R_ (110–142 ppm) + C_Ar–O, N_ (142–156 ppm)/total peak area (0–230 ppm])*100

^3)^ Aliphaticity: Aliph = (100 –Arom)

^4)^ HI = [C_Alk–H, R_ (0–45 ppm) + C_Alk–O, N_ (45–60 ppm) + C_Ar–H, R_ (110–142 ppm) + C_Ar–O, N_ (142–156 ppm)] / [C_Alk–O_ (60–91 ppm) + C_Alk–di–O_ (91–110 ppm) + C_COO–H, R_ (156–186 ppm) + C_C = O_ (186–230 ppm)]

The relative intensities of the different carbon chemical shifts differed in the ten samples. The spectral data were divided into eight regions, as shown in Table 6, to quantify the carbon functional groups by the method of García *et al* [37]. Oxidation with H_2_O_2_ can change the content of different carbon types depending on the oxidizing conditions. The C_Alk–O, N_ and C_Ar–H, R_ contents of HA were higher than those of the OHAs. However, the C_Alk–di–O_, C_Ar–O, N_ and C_COO–H, R_ contents of HA were lower than those of the OHAs. The C_Alk–H, R_, C_Alk–O_ and C_Alk–di–O_ contents of OHA5 were higher than those of HA and other OHAs. The C_COO–H, R_ content of OHA2, OHA6 and OHA7, which were prepared at 60°C, was higher than that of the others. Additionally, these results showed that the oxidation process has no significant effect on the aromaticity and aliphaticity of HA. However, the hydrophobicity index (HI) of HA is higher than that of the OHAs.

Fig 4 shows the PCA results with 90.91% of the total variance explained based on the relative number of carbon types for each kind of humic acids. The OHAs were clustered and different from HA. OHA2 and OHA6 were clustered in PC1 (59.30%) because of the predominance of substituted aromatic and carboxyl carbon groups. OHA1, OHA5, OHA8 and OHA9 were clustered with negative values because of the predominance of aliphaticity and di–O–alkyl carbon. OHA3 and OHA4 were clustered in PC2 (31.61%) because of the predominance of aliphatic and unsubstituted aromatic carbon groups. OHA7 was dominated by carbonyl and aromaticity carbon.

### Relationship between the elemental content and atomic ratios, E2/E3 and E4/E6 of the humic acids and the oxidation conditions

A MANOVA was conducted to determine the relationship between the properties of humic acids and the preparation conditions. The results from SPSS are shown in Table 7, which is slightly modified for easier reading. The statistical test Wilks’ Lambda statistics are shown. The values can be converted to an F statistic, which can then be used to calculate a p value, and these values are displayed in Table 7. The MANOVA test statistics for the data are statistically significant (p< 0.05). This result shows that the null hypothesis has been rejected, and the hydrogen peroxide concentration, liquid-to-solid ratio, time and temperature have a statistically significant relationship with the properties of humic acids. In this hypothesis test, the value for temperature is the largest which followed by hydrogen peroxide concentration, liquid-to-solid ratio and time, indicating that temperature has the largest contribution to the model. Temperature has the largest partial η^2^ and the largest contribution to the difference, followed by the hydrogen peroxide concentration, liquid-solid ratio, and time. Thus, among the preparation conditions, temperature is the most important factor affecting the properties of humic acids.

**Table 7:** Relationship between the elemental content and atomic ratios, E2/E3 and E4/E6 of humic acids and the oxidation conditions.

Factors	Value (Statistic)	F	*p* value	Partial η^2^
concentration of hydrogen peroxide	0.052	3.746	0.003	0.771
liquid-to-solid ratio	0.064	3.293	0.006	0.748
time	0.074	2.985	0.010	0.729
temperature	0.036	4.706	0.001	0.809

## Discussion

### Effects of oxidizing conditions on the structural characteristics of humic acids

The structural pattern of the humic acids derived from weathered coal according to the ^13^C–CP/MAS–NMR assay was comparable with that of humic acids from weathered coals [32, 38], lignite [20–21, 39–40], and even composted wastes [41–42]. This result demonstrated the samples in the present investigation well represent a general humic acids structural pattern. On average, the oxidation with H_2_O_2_ decreased the C content, increased the O and O/C contents. Among all the treatments samples, OHA3 and OHA4 showed relatively higher C contents than the others. More importantly, HA had a higher C content than fulvic acid extracted from soil, suggesting that the C content might be an indicator of molecular weight. OHA3 and OHA6 showed the highest and lowest C/H contents, respectively and high C/H ratios are thought to indicate high stability of humus and large degree of condensed structures [43]. In addition, OHA6 and OHA3 had the highest and lowest O/C ratios, respectively, indicating that OHA6 had higher contents of carbohydrates and carboxylic acids [43]. Oxidation with H_2_O_2_ can increase the oxygen–bearing group content of humic acids, possibly resulting from the oxidative reactions with hydrogen peroxide. It has been demonstrated by others that the E2/E3 and E4/E6 values are negatively correlated with the aromaticity, condensation and molecular weight of humic acids [33,44]. All the OHAs had a higher E4/E6 ratio than the HAs directly extracted from the Chinese weathered coal. This difference indicated that H_2_O_2_ oxidation might decrease the molecular weight of humic acids. In addition, the E4/E6 ratio of HAs in this work was different from that reported in previous studies. In the current situation, the E4/E6 ratios of HA and OHAs were in the range of 3.1–3.69, while those in other investigations were in the range of 4.00–7.00 [45–46], and this difference suggest that the materials in this research contained more condensed ring structures and had a higher molecular weight.

The biological activity of humic acids is determined by various functional groups, which also reflect the origin materials and mechanism of formation. In addition, chemical modification can also change the functional composition and structure of humic acids. In this research, oxidation with H_2_O_2_ can change the content of different carbon types depending on the oxidizing conditions. Some kinds of OHAs have more aromatic carbon and carboxyl/carbonyl carbon groups than HA [47], while, the others have fewer groups. A previous study showed that chemical modification by H_2_O_2_ can increase the contents of carbonyl and carboxyl carbon groups, and groups containing oxygen atoms. Correspondingly, the contents of C_Alk–H, R_, C_Alk–O_, and C_Alk–O, N_ are reduced. The difference between the results from our work and previous research may be due to the reaction conditions. In this research, we attempted to obtain humic acids with various functional groups to further investigate their application in nature and technological processes to enable more effective use of these materials.

### Potential utilization of humic acids with different functional groups in agriculture

Humic acids contain various functional groups, a variety of trace elements and other beneficial components [3–4]. Due to the presence of various active functional groups, humic acids have the acidic, hydrophilic, interfacial activity, cation exchange, complexation, adsorption and dispersion function [48]. The OHAs oxidized at 60°C showed a higher content of carboxyl/carbonyl carbon groups than the original humic acids. Previous studies have shown that HAs with higher carboxyl group contents can perform better in chemical industry applications, such as ion exchange and wastewater purification. Our research found that OHAs had lower contents of aromatic carbon and higher contents of O–aromatic carbon and carboxyl carbon than HA. In addition, previous studies have showed that O–aromatic carbon structures and carboxyl groups can stimulate plant growth, indicating the great potential of OHAs for use in agriculture [22–23, 49]. The hydrophobicity index of HA was higher than that of the OHAs. Canellas *et al* [50] showed that lateral root emergence is mostly related to the hydrophobicity index and hydrophilic carbon, and the content of hydrophobic carbon in humic acids is negatively correlated with the induction of lateral root hair.

**Fig 5:**
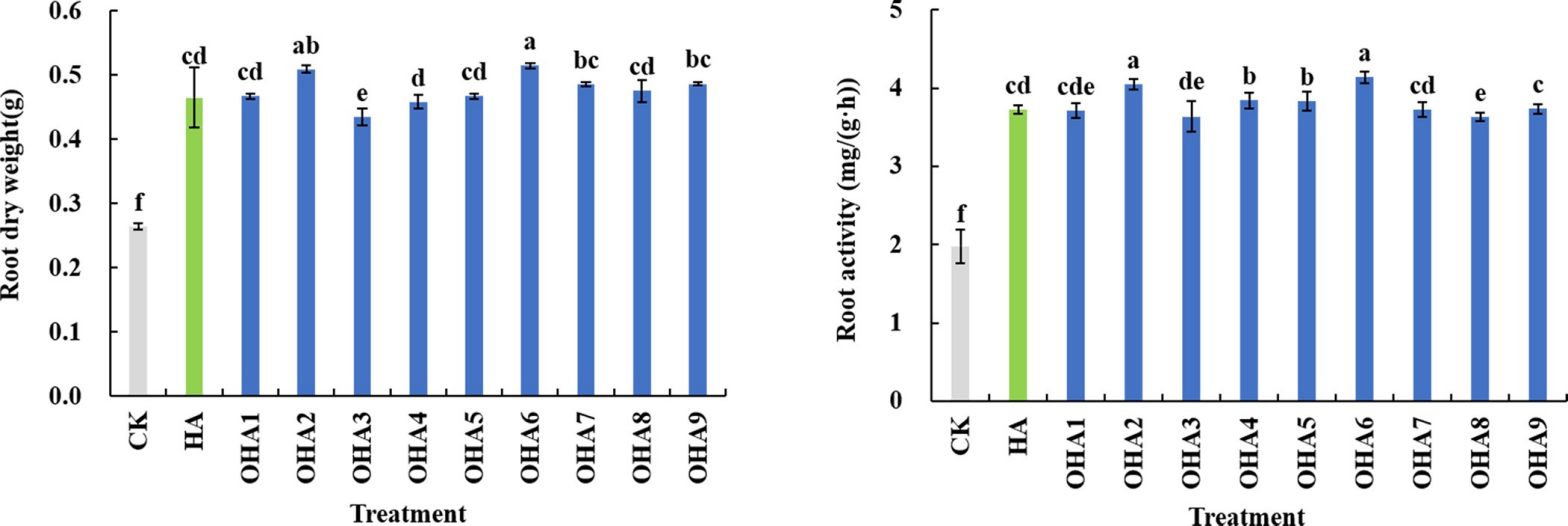
Root dry weight and root activity of maize affected by HA and OHAs. CK, no humic acids; HA, original humic acids which is derived from Chinese weathered coal; OHA1-OHA9, humic acids under different oxidation conditions.

To investigate the effects of different kinds of OHAs in agriculture, an experiment was conducted to explore the effect of HA and OHAs on maize roots. The dry weight and root activity of maize root were measured. OHA6 which had the highest contents of O, O/C, highest E4/E6 ratio, relatively high carboxyl carbon content and lowest aromatic carbon content, showed the optimal effect for promoting maize. OHA3, which had the highest contents of C and C/H, had the lowest effect on maize root growth (Fig 5). Further work is underway to investigate the regulatory mechanism of humic acids on maize roots.

## Conclusions

H_2_O_2_ oxidation altered the structure and composition of humic acids derived from Chinese weathered coal. On average, H_2_O_2_ oxidation decreases the H and N contents and increases the O content. H_2_O_2_ oxidation can decrease the hydrophobicity index of humic acids. Among the various studied preparation conditions, temperature is the most important factor affecting the properties of humic acids. The prepared OHAs with different characteristics have potential to serve as functional materials for further study in agriculture and other industries. An investigation into the application of HAs and OHAs to promote maize roots growth is currently underway.
